# Evaluating the value of ^18^F-PSMA-1007 PET/CT in the detection and identification of prostate cancer using histopathology as the standard

**DOI:** 10.1186/s40644-023-00627-x

**Published:** 2023-11-03

**Authors:** Liang Luo, Anqi Zheng, Ruxi Chang, Yunxuan Li, Jungang Gao, Zhuonan Wang, Xiaoyi Duan

**Affiliations:** PET/CT Center, The First Affiliated Hospital of Xi’an Jiaotong University, No. 277 Yanta West Road, Shaanxi Province, Xi’an, 710061 China

**Keywords:** Prostate cancer, Prostate-specific membrane antigen, PET/CT, SUVmax cutoff

## Abstract

**Background:**

Prostate-specific membrane antigen (PSMA) PET/CT is a highly regarded radionuclide imaging modality for prostate cancer (PCa). This study aimed to evaluate the diagnostic performance of ^18^F-PSMA-1007 PET/CT in detecting intraprostatic lesions of PCa using radical prostatectomy (RP) specimens as a reference standard and to establish an optimal maximum standardized uptake value (SUVmax) cutoff for distinguishing between PCa and non-PCa lesions.

**Methods:**

We retrospectively collected 117 patients who underwent ^18^F-PSMA-1007 PET/CT before RP. The uptake of the index tumor and contralateral non-PCa lesion was assessed. Histopathology of RP specimens was used as the gold standard. Kappa test was used to evaluate the consistency of preoperative PSMA PET/CT staging and postoperative pathological staging. Finally, an SUVmax cutoff value was identified by receiver operating characteristic (ROC) curve analysis to distinguish PCa lesions from non-PCa lesions. A prospective cohort including 76 patients was used to validate the results.

**Results:**

The detection rate of ^18^F-PSMA-1007 PET/CT for prostate cancer was 96.6% (113/117). ^18^F-PSMA-1007 had a sensitivity of 91.2% and a positive predictive value (PPV) of 89.8% for the identification of intraprostatic lesions. The consistency test (Kappa = 0.305) indicated poor agreement between the pathologic T-stage and PSMA PET/CT T-stage. Based on ROC curve analysis, the appropriate SUVmax to diagnose PCa lesions was 8.3 (sensitivity of 71.3% and specificity 96.8%) with an area under the curve (AUC) of 0.93 (*P* < 0.001). This SUVmax cutoff discriminated PCa lesions from non-PCa lesions with a sensitivity of 74.4%, a specificity of 95.8% in the prospective validation group.

**Conclusions:**

^18^F-PSMA-1007 PET/CT demonstrated excellent performance in detecting PCa. An optimal SUVmax threshold (8.3) could be utilized to identify lesions of PCa by ^18^F-PSMA-1007 PET/CT.

**Trial registration:**

ClinicalTrials.gov Identifier: NCT04521894, Registered: August 17, 2020.

## Background

Prostate cancer (PCa) is a prevalent malignancy of the male genitourinary system and the fifth leading cause of cancer-related mortality in men [1]. The accurate staging of this disease is critical for treatment planning and prognosis. Prostate-specific membrane antigen (PSMA) is a type II membrane glycoprotein that exhibits overexpression in PCa cells while being either absent or moderately expressed in most hyperplastic and benign tissues [2, 3]. PSMA positron emission tomography/computed tomography (PET/CT) is a highly regarded radionuclide imaging modality for PCa that is increasingly being used for initial staging because its diagnostic accuracy is higher than that of conventional imaging [4–6]. However, distinguishing between benign and malignant prostate lesions based on visual information from PSMA PET/CT can pose a challenge at times.

Currently, several radiolabeled PSMA ligands are available for PSMA PET imaging, mainly ^68^ Ga-coupled PSMA ligands and ^18^F-coupled ligands [7].^18^F-PSMA-1007 is a novel radiotracer and widely used ^18^F-PSMA ligand that exhibits high accuracy in detecting primary PCa lesions [8]. In addition, ^18^F-PSMA-1007 is excreted primarily in bile rather than the urinary tract for better evaluation of the prostatic bed [9]. Previous investigations have primarily focused on the invasion of lymph nodes and the occurrence of distant metastases of ^18^F-PSMA-1007 [10, 11]. Only a limited number of studies have examined the efficacy of ^18^F-PSMA-1007 as a primary T staging modality using radical prostatectomy (RP) specimens as a reference. Kesch et al. [12] demonstrated in a study involving 10 patients with biopsy-confirmed high-risk PCa that ^18^F-PSMA-1007 had a sensitivity and specificity of 71% and 81%, respectively, compared to RP specimens. Further research with larger sample sizes is imperative.

Although the criteria of PSMA-RADS, PROMISE, and E-PSMA have improved lesion classification and interpretation based on specific imaging features, disagreements exist in clinical practice regarding the evaluation of ^18^F-PSMA-1007 PET/CT results due to a lack of quantitative standards [13]. Consensus statements regarding PSMA PET/CT have highlighted the pressing need for a defined cutoff value of maximum standardized uptake value (SUVmax) [14]. The use of SUVmax is appropriate for the diagnosis of primary PCa due to its significant correlation with PSMA expression [15]. However, there are few studies concerning ^18^F-PSMA-1007 that focused on the threshold value for SUVmax to discriminate PCa from non-PCa lesions.

Therefore, the primary objective of this study was to evaluate the diagnostic accuracy of ^18^F-PSMA-1007 PET/CT in detecting intraprostatic lesions of PCa with RP specimens as the reference standard. The secondary goal was to identify a threshold SUVmax for distinguishing between PCa and non-PCa lesions.

## Material and methods

### Patient population

**Fig. 1:**
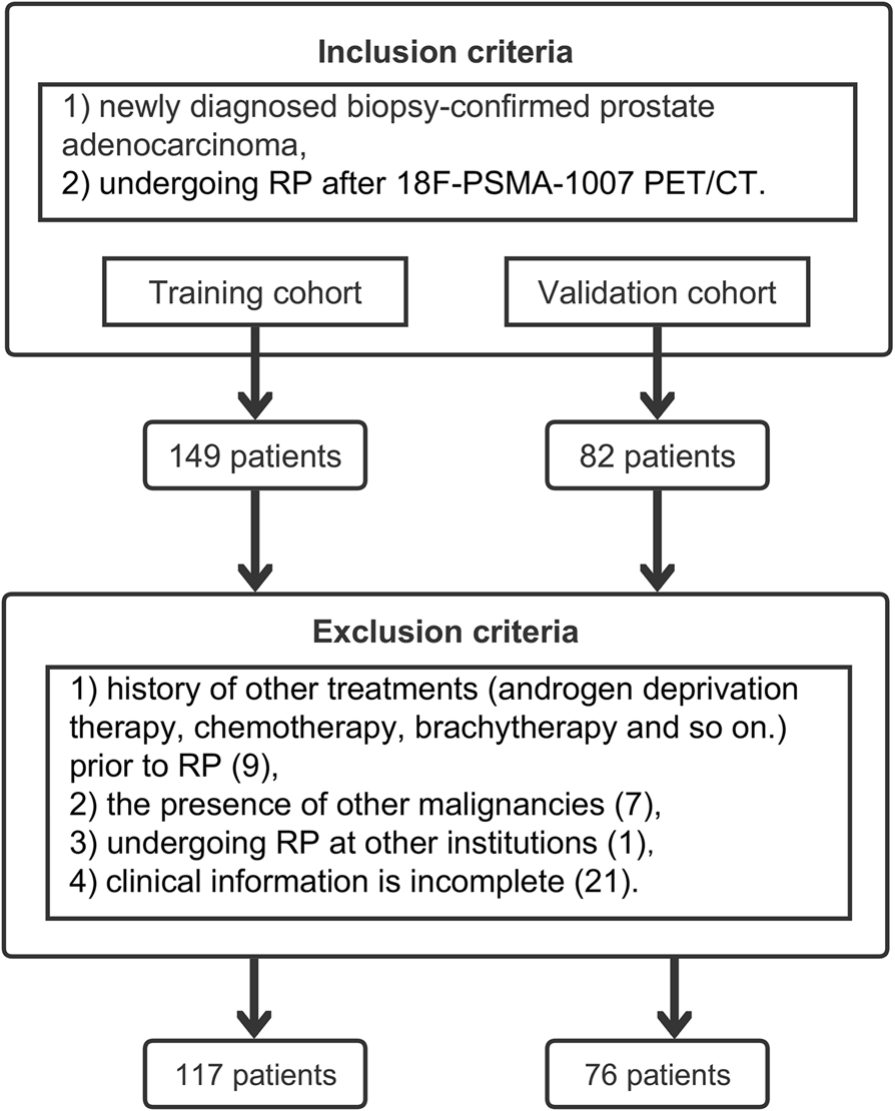
Study flowchart. RP: radical prostatectomy

Data from patients who underwent ^18^F-PSMA-1007 PET/CT at a large tertiary care hospital in China between July 2020 and September 2022 were retrospectively collected. The inclusion criteria were 1) newly diagnosed biopsy-confirmed prostate adenocarcinoma and 2) undergoing RP after ^18^F-PSMA-1007 PET/CT. The exclusion criteria were as follows: 1) history of other treatments (androgen deprivation therapy, chemotherapy, brachytherapy and so on.) prior to RP, 2) the presence of other malignancies, 3) undergoing RP at other institutions, and 4) clinical information is incomplete. An independent validation group was acquired from a prospective research trial (NCT04521894) in the same center between October 2022 and August 2023 (Fig. 1). This study was approved by the hospital ethics committee, and all participating patients provided written informed consent.

### ^18^F-PSMA-1007 manufacture and image acquisition

Radiolabeling was performed using a fully automated radiopharmaceutical synthesis device based on a modular concept (MINItrace; GE Healthcare, USA). Over 99% radiochemical purification yield ^18^F-PSMA-1007 was obtained and examined by both radiation layer chromatography and high-performance liquid chromatography analysis. All ^18^F-PSMA-1007 PET/CT data were acquired using a PET/CT scanner (Gemini 64TF, Philips, The Netherlands) at a single location. Patients received an intravenous injection of ^18^F-PSMA-1007 (3.7 MBq/kg body weight) and underwent PET and CT scans 90 min after the injection. Low-dose CT scans from the head to the proximal thighs (pitch 0.8 mm, 60 mA, 140 kV [peak], tube single turn rotation time 1.0 s, and 5-mm slice thickness) for PET attenuation were acquired (pitch 0.8 mm, automatic mA, 140 kV [peak], and 512 × 512 matrix). Whole-body PET scans were performed in three-dimensional mode (emission time: 90 s per bed position, scanned at a total of 7–10 beds), as described in our previous study [16].

### Image analysis

In the first analysis, two experienced board-certified nuclear medicine specialists (X.D. and Z.W.) retrospectively interpreted all ^18^F-PSMA-1007 PET/CT scans using Fusion Viewer software in the Extended Brilliance Workstation (EBW, Philips, Netherlands). The presence of unilateral (T2a-T2b) and bilateral (T2c) intraprostatic disease and seminal vesicle invasion (T3b) were assessed. Only basic patient characteristics are known to the specialist when analyzing the images. In the second analysis, Z.W. using RP specimens as standard, adapted an automatically drawn 3-dimensional volume of interest with the software to calculate the maximum standardized uptake value (SUVmax) of PCa lesions and manually drew a 1.5-cm-diameter circular region of interest in the contralateral non-PCa region to measure SUVmax (if possible).

### Histopathology

All slices (tissue sections of 6 mm, histologic sections cut at 4 μm) of all RP specimens were evaluated and interpreted according to the International Society of Urological Pathology (ISUP) criteria by an experienced pathologist. The location of the index tumor (defined as the area where the tumor showed its maximum diameter on each side of the prostate) [17], extracapsular extension (T3a), and seminal vesicle invasion, as well as the Gleason score and ISUP grade, were assessed. The specialist was blinded to both the clinical evaluation of the samples and the ^18^F-PSMA-1007 PET/CT results.

### Statistical analysis

Patient demographics were analyzed descriptively. For index tumor localization analysis, each prostate was divided into left and right segments. The PET/CT scan was considered concordant with the histopathology findings if the index tumor lesion was on the same segment, that is, on the same side. Kappa test was used to evaluate the consistency of preoperative PSMA PET/CT staging and postoperative pathological staging. When Kappa is greater than 0.75, it shows a high degree of consistency; when Kappa is between 0.40 and 0.75, it suggests good consistency; and when Kappa is less than 0.40, it indicates poor agreement. Due to the low spatial resolution of PET and the difficulty of discriminating between prostatic lesions and the capsule on CT, identifying extraprostatic extension (T3a) on PET/CT is challenging, patients with pathologically confirmed T3a were excluded in the tumor stage analysis. The true positive, false positive, true negative, and false negative results of pathology and PSMA PET/CT are described with a fourfold table. Sensitivity, specificity, positive predictive value (PPV), and negative predictive value (NPV) were calculated. The Kruskal‒Wallis test was used to assess the associations of the ISUP grade and PSA groups with the SUVmax of the index tumor, with a Mann‒Whitney U test used as a post hoc test. A receiver operating characteristic (ROC) curve was generated to determine the classification performance of SUVmax in distinguishing PCa and non-PCa lesions. The cutoff value of SUVmax was identified by Youden’s index (sensitivity + specificity – 1) to maximize sensitivity and specificity.

## Results

### Demographics

A total of 149 biopsy-confirmed PCa patients underwent ^18^F-PSMA-1007 PET/CT and RP between July 2020 and September 2022. Of those, 117 patients (group A) were included in the final analysis of this study, with a mean age of 69 y (range, 48–85 y). The median time interval between ^18^F-PSMA-1007 PET/CT and RP was 7 days (IQR, 5–10 d). In the prospective validation cohort (group B), 76 patients from October 2022 to August 2023 at the same center were recruited for SUVmax cutoff analysis. Patient characteristics are summarized in Table 1.

**Table 1:** Clinical characteristics of patients

Characteristics	Group A	Group B
**Age (y), mean ± SD**	69 ± 6.9	71 ± 8.2
**Days from PET to surgery, median (IQR)**	7 (5–10)	8 (6–13)
**PSA at diagnosis (n)**		
< 10	21	18
10–20	49	34
> 20	47	24
**T stage based on PSMA PET/CT (n)**		
< = T2b	21	18
> = T2c	96	58
**Pathological T stage (n)**		
T2a/T2b	15	12
T2c	18	6
T3a	61	50
T3b	23	8
**ISUP (n)**		
1	7	3
2	16	24
3	40	18
4	24	16
5	30	15

*SD* Standard deviation, *IQR* Interquartile range, *ISUP* International Society of Urological Pathology

**Fig. 2:**
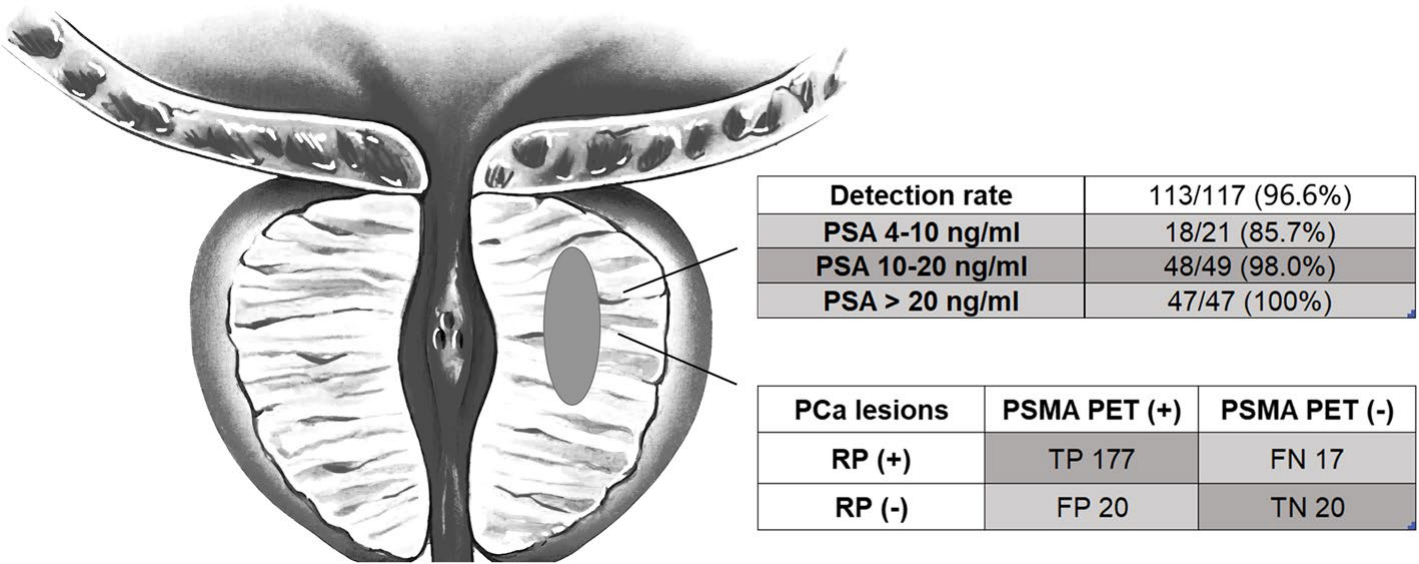
Diagnostic performance of ^18^F-PSMA-1007 PET/CT in the detection of prostate cancer lesions using histopathology as the standard. RP: radical prostatectomy

Of the 117 patients with biopsy-confirmed PCa, ^18^F-PSMA-1007 PET/CT was positive in 113 (96.6%) patients. The detection rates were 85.7%, 98.0% and 100% for PSA levels between 4–10 ng/ml, 10–20 ng/ml and above 20 ng/ml, respectively (Fig. 2).

### Lesion-based and tumor stage analysis

In total, 194 tumor lesions were detected by histopathology and 197 tumor lesions were identified by PSMA PET/CT. 177 of the 197 lesions were classified as true PSMA PET-positive, whereas the remaining 17 were considered as false negatives (Fig. 2). The sensitivity, specificity, PPV and NPV of intraprostatic lesions identification on ^18^F-PSMA-1007 PET/CT were 91.2%, 50%, 89.8% and 54.1%, respectively.

After excluding the 61 patients with pathologically confirmed T3a, the correlation between the pathologic T stage and PSMA PET/CT T stage is summarized in Table 2. The PSMA PET/CT T stage agreed with the pathological T stage in 43 of the 56 (76.8%) patients, and the consistency test (Kappa = 0.305) revealed a low level of agreement.

**Table 2:** Comparison of PSMA PET/CT T stage results and pathological T stage results

PSMA PET/CT staging	Pathological diagnostic staging		Total
	** < = T2b**	** > = T2c**	
**< = T2b**	5	3	8
**> = T2c**	10	38	46
**Total**	15	41	56

### SUVmax is correlated with PSA and ISUP grade

**Fig. 3:**
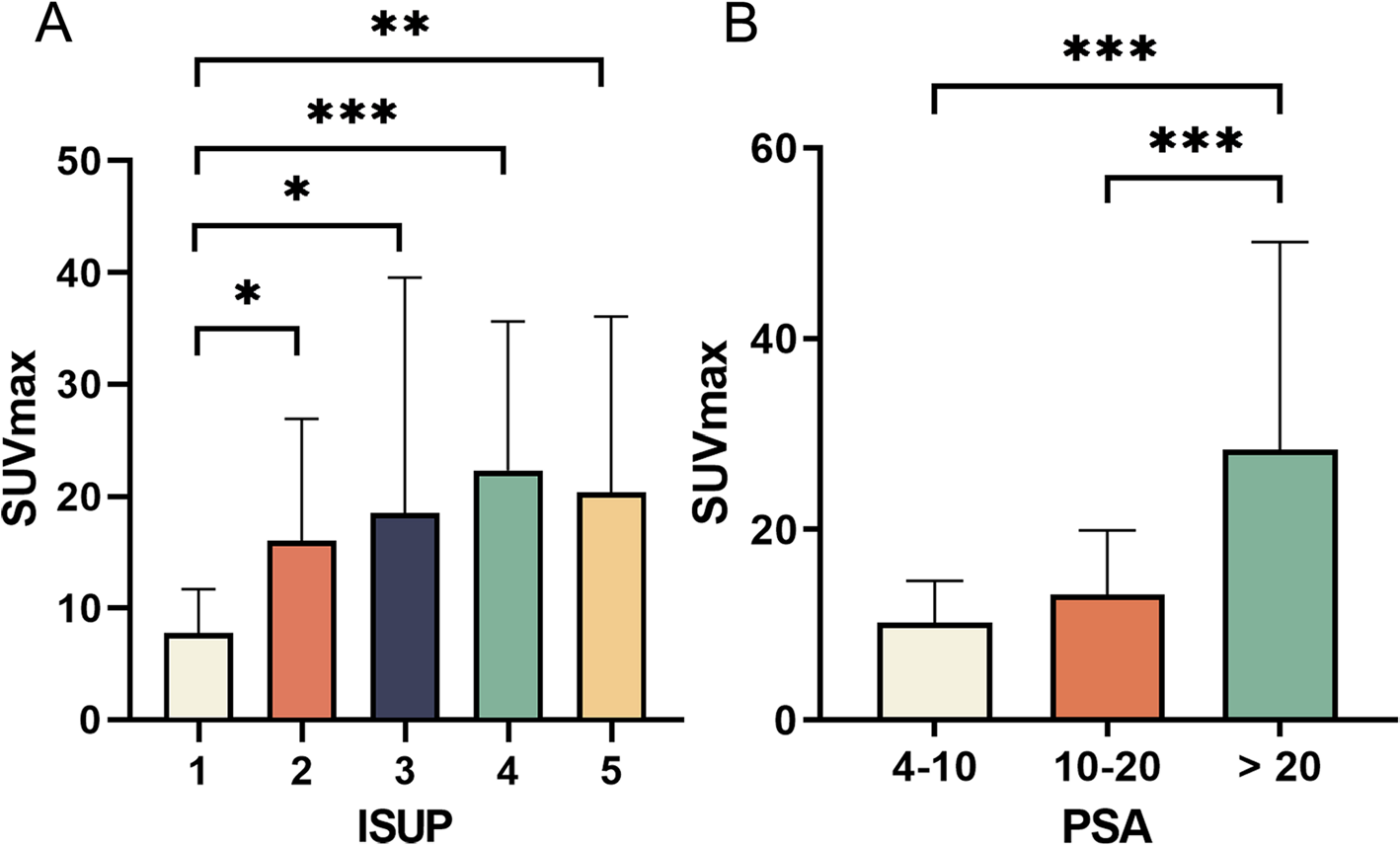
Histograms for SUVmax of different (**A**) ISUP grades and (**B**) PSA levels for the index tumors

By comparing the SUVmax of index tumors at different PSA levels and ISUP grades, Fig. 3 demonstrated significant overall differences (*P* < 0.001 and *P* = 0.005, respectively). The post hoc analysis revealed a positive correlation between SUVmax of the index tumor and serum PSA level and demonstrated that ISUP grade 1 had significantly lower SUVmax than grades 2–5, with no significant differences observed among other groups.

### An SUVmax cutoff value to discriminate PCa and non-PCa lesions

**Fig. 4:**
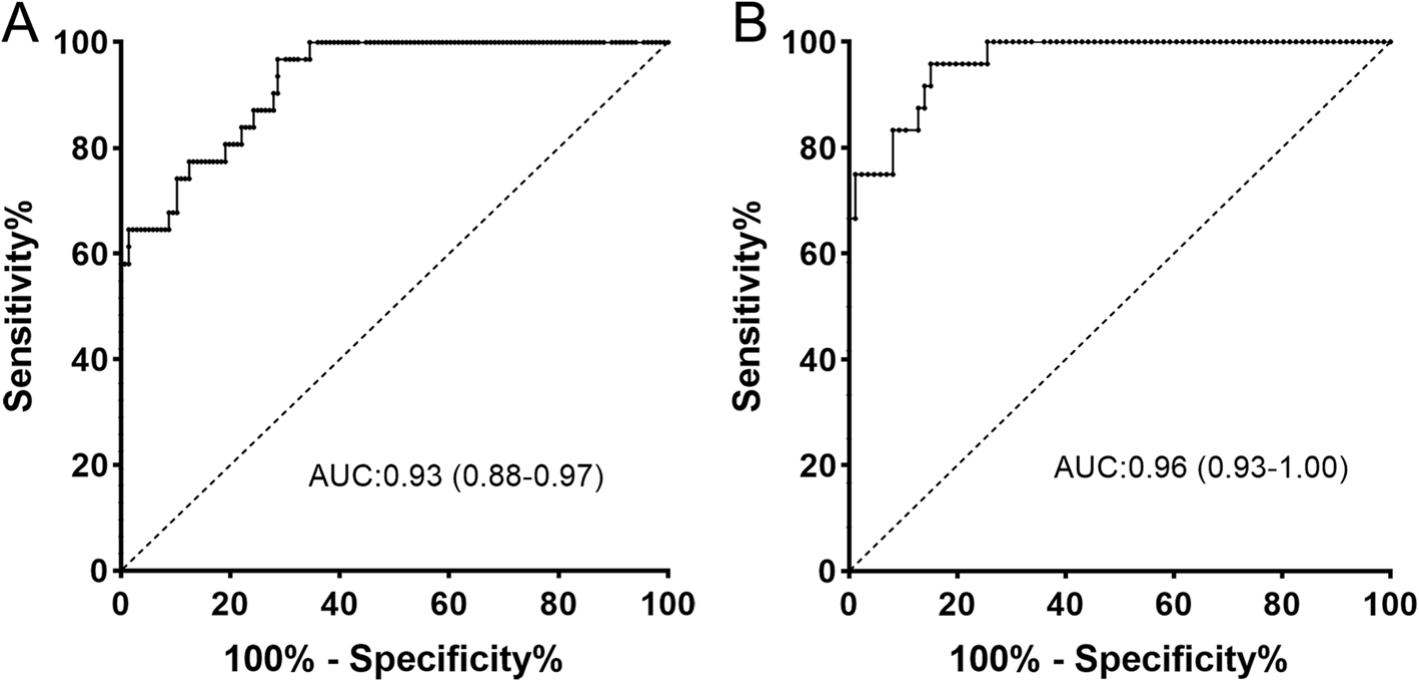
ROC curve analysis of SUVmax to differentiate prostate cancer and non-prostate cancer lesions. **A** The SUVmax cutoff value of 8.30 yielded a sensitivity of 71.3% and a specificity of 96.8% in the training cohort (AUC = 0.93). **B** The cutoff of 8.30 achieved a sensitivity of 74.4% and a specificity of 95.8% in the prospective validation cohort

In the training cohort, the median SUVmax of PCa and non-PCa was 12.2 and 3.6, respectively. ROC curve analysis (Fig. 4A) demonstrated that the best SUVmax discriminated between PCa and non-PCa lesions with an area under the curve (AUC) of 0.93 (95% confidence interval: 0.88–0.97, *P* < 0.001). An SUVmax cutoff value of 8.3 was identified as the optimal threshold based on Youden’s index (sensitivity 71.3% and specificity 96.8%). In the prospective validation cohort, a total of 110 lesions were identified by PSMA PET/CT. The SUVmax cutoff value of 8.3 achieved a sensitivity of 74.4%, a specificity of 95.8% (AUC: 0.96, Fig. 4B), and 64 (SUVmax > 8.3) of 110 lesions (58.2%) were concordant to the pathological findings.

## Discussion

In this study, ^18^F-PSMA-1007 PET/CT demonstrated high sensitivity and PPV for detecting intraprostatic lesions in patients with PCa, using RP specimens as a reference standard. The accurate identification of index tumors in most patients suggested that ^18^F-PSMA-1007 PET/CT is a reliable method for PCa detection. However, we identified a small proportion of false-negative lesions on ^18^F-PSMA-1007 PET/CT, which may result in missed diagnoses during clinical practice. A previous study showed that tumor lesions with less than 90% PSMA-positive cells may not be detected on PSMA PET/CT scans due to inadequate PSMA expression [18]. Our study also observed a proportion of false-positive lesions. It is worth noting that PSMA can be expressed in various conditions other than prostate cancer, including benign prostatic tissue, albeit at a lower intensity than in prostate cancer cells [19].

We observed a positive correlation between the SUVmax of the index tumor and PSA level, with the group of PSA levels ranging from 4–10 ng/ml demonstrating the lowest SUVmax. This finding is consistent with that reported by a retrospective cohort study involving 194 patients who underwent ^18^F-PSMA-1007 PET/CT [20], which indicated a significant correlation between a higher SUVmax of the primary prostate tumor and rising PSA levels. In addition, the detection performance of ^18^F-PSMA-1007 PET/CT in patients with low PSA levels, particularly those with PSA levels below 10 ng/ml, was inferior to that in patients with high PSA levels. In future studies, we will conduct a prospective study to evaluate the clinical utility of ^18^F-PSMA-1007 in patients with suspected prostate cancer within the PSA gray zone [21].

In our study, an SUVmax cutoff value of 8.3 in the training and validation groups achieved a specificity of 96.8% and 95.8% for distinguishing between PCa and non-PCa lesions, respectively, indicating that this cutoff value could be instrumental in guiding active patient management, such as surgery or systematic therapy. However, since the exact measurement of SUVmax may vary between different PET/CT acquisitions and protocols [22], SUVmax alone was deemed insufficient for the diagnosis of patients with PCa. Instead, the cutoff value for SUVmax proposed in this study serves as a reference for other centers and primarily guides future prospective studies at our institution. The sensitivity of the cutoff value of SUVmax both in the training and validation groups was low, implying that the cutoff value of 8.3 may result in missed diagnosis of some lesions. As a result, lesions with low SUVmax should be comprehensively analyzed in combination with other clinical and imaging information.

According to the D’Amico risk classification, the American Joint Commission on Cancer Staging T stage is included in the standards that differentiate the risk of PCa [23]. T2b and T2c, in particular, are used to differentiate between intermediate- and high-risk PCa. As a result, this study investigated the application of PET/CT for tumor stages (< = T2b and >  = T2c). However, due to the low spatial resolution of PET and the difficulty of discriminating between prostatic lesions and the capsule on CT, identifying extraprostatic extension (T3a) on PET/CT is challenging. A prior study indicated that the diagnostic accuracy of ^18^F-PSMA-1007 PET/CT for detecting extraprostatic extension was 57% and poor reliability was found among PSMA PET/CT readers [24]. Hence, patients with pathologically confirmed T3a were excluded from this study to avoid the influence of T3a on T staging. Our study showed poor consistency between PSMA PET T stage and pathological T stage. However, a previous study [25] demonstrated the favorable performance of multiparametric MRI in localizing intraprostatic lesions of PCa, which rendered it a viable option for T-staging assessment. Therefore, it is postulated that PET/MRI or a combination of multiparametric MRI along with ^18^F-PSMA-1007 PET/CT may have the potential to improve local staging.

To the best of our knowledge, some studies have evaluated the efficacy of ^18^F-PSMA-1007 PET/CT for the detection of PCa in comparison with RP specimens. Kesch et al. [12] performed ^18^F-PSMA-1007 PET/CT in 10 patients with biopsy-confirmed PCa and reported a PPV of 83% and an NPV of 68% for total agreement. Trägårdh et al. [26] studied 39 patients who underwent RP, and the results showed that ^18^F-PSMA-1007 PET/CT detected 62/118 intraprostatic tumor lesions (55 true-positive and 7 false-positive), while 63 tumor lesions were classified as false-negative. Compared to the abovementioned studies, our investigation was the largest to date with 117 patients to evaluate the detection performance of ^18^F-PSMA-1007 PET/CT using RP specimens as a standard reference. Additionally, we conducted an analysis on the efficacy of PSMA PET/CT in T staging and established a cutoff value for SUVmax that might aid in distinguishing between PCa and non-PCa lesions.

There are two main limitations to this study. First, due to the retrospective collection of pathological data, prostate lesions were only divided into left and right segments rather than into twelve or twenty-four segments [12, 26]. However, as the goal of this study was to evaluate the diagnostic efficacy of ^18^F-PSMA-1007 PET/CT for PCa, detailed tumor localization may not significantly impact the results. Second, given that the majority of tumor lesions were pathologically staged as T3a and PET/CT is not suitable for identifying extraprostatic extension, the results of diagnostic efficacy for T-staging may be more or less influenced. Therefore, a larger sample size is required to validate the results.

## Conclusion

By using radical prostatectomy specimens as standards, ^18^F-PSMA-1007 PET/CT demonstrates excellent performance in detecting the index tumor in prostate cancer. We identified and validated an optimal SUVmax cutoff value (8.3) to identify prostate cancer lesions. This cutoff value may have the potential to aid in the diagnosis of patients with prostate cancer using ^18^F-PSMA-1007 PET/CT.

## Data Availability

The datasets used and/or analyzed during the current study are available from the corresponding author on reasonable request.

## Abbreviations

PCa: Prostate cancer
RP: Radical prostatectomy
SUVmax: Maximum standardized uptake value
ROC: Receiver operating characteristic
PPV: Positive predictive value
NPV: Negative predictive value
PSMA: Prostate-specific membrane antigen
ISUP: International Society of urological pathology
AUC: Area under curve
